# Factors Affecting Grazing and Rumination Behaviours of Dairy Cows in a Pasture-Based System in New Zealand

**DOI:** 10.3390/ani12233323

**Published:** 2022-11-28

**Authors:** Muhammad Wasim Iqbal, Ina Draganova, Patrick C. H. Morel, Stephen T. Morris

**Affiliations:** School of Agriculture and Environment, College of Sciences, Massey University, Private Bag 11-222, Palmerston North 4442, New Zealand

**Keywords:** automatic behaviour monitoring, grazing behaviour, rumination behaviour, pasture-based system, individual animal data

## Abstract

**Simple Summary:**

Understanding the trends in grazing and rumination behaviours and their variation can provide farmers with information about the health status, physiological state, productivity, and welfare of the animal. Considering this, we studied variations in grazing and rumination times in grazing dairy cows due to breed, lactation year, breeding worth, days in milk of the individual cow, and season and supplementary feeds. AfiCollar device was used to monitor and record the behaviours of grazing cows affiliated with Holstein-Friesian, Jersey, and KiwiCross breeds in different years of lactation during different seasons over three years. We found grazing time and rumination time varied among the individual cows, during different seasons (or stages of lactation), and when cows were fed with different supplements. Jersey cows, and in general, the cows in the first year of lactation relatively grazed for a longer period. Grazing time and rumination time were longer at the start of lactation in spring and shorter at the end of lactation in autumn. These findings could contribute to improving the measures for pasture management, and additional feed supply for a mixed herd comprising Jersey, Holstein-Friesian and KiwiCross breeds in different years of lactation during different seasons over the lactation period.

**Abstract:**

This study investigated the variation in daily time spent grazing and rumination in spring-calved grazing dairy cows (n = 162) of three breeds, Holstein-Friesian (HFR), Jersey (JE), and KiwiCross (KC) with different breeding worth index, and in different years of lactation (1st, 2nd, 3rd, 4th). The cows were managed through a rotational grazing system and milked once a day at 05:00 a.m. The cows grazed mainly pasture and received supplementary feeds depending on the season. Automated AfiCollar device continuously monitored and recorded grazing time and rumination time of the individual cows throughout the lactation period for three study years (Year-1, Year-2, Year-3) with 54 cows per year. A general linear mixed model fitted with breed × lactation year with days in milk (DIM), breeding worth (BW) index value, individual cow, season, and feed, and their interactions was performed in SAS. Variance partitioning was used to quantify the effect size of study factors and their interactions. Individual cows, DIM, and BW (except Year-3) had effects on grazing and rumination times throughout the study years. Grazing time and rumination time were different for different seasons due to varying supplementary feeds. Grazing time varied among breeds in Year-2 and Year-3, and among lactation years only in Year-1. Although rumination time differed among breeds in Year-3, it remained the same within different lactation years. Grazing time and rumination time had a negative relationship with each other, and their regression lines varied for different seasons. The total variance explained by the model in grazing time was 36–39%, mainly contributed by the individual cow (12–20%), season (5–12%), supplementary feed (2–6%), breed (1–5%), and lactation year (1–6%). The total variance explained in rumination was 40–41%, mainly contributed by the individual cow (16–24%), season (2–17%), supplementary feed (1–2%), breed (2–8%), and lactation year (~1%). These findings could contribute to improving the measures for feed resource management during different seasons over the lactation period for a mixed herd comprising JE, HFR and KC breeds in different years of lactation.

## 1. Introduction

Grazing and rumination are predominant behaviours and provide key knowledge about the satiety needs, and how those demands are addressed, hence playing a pivotal role in the nutrition of grazing ruminants. Grazing regulates intake from the grazed herbage and rumination determines the digestive efficiency, health, and well-being of the animal [1,2]. Grazing dairy cows allocate 90–95% of their daily time to grazing, ruminating, and resting [3]. They normally exhibit four major periods of grazing occupying a total of seven to eight hours and a similar period for rumination; they spend the rest of the day resting or idling [4]. The times spent grazing and ruminating are the main indicators for pasture management and animal welfare [5]. In addition, behavioural decisions made by animals result in variations in intake rate [6] and affect animals’ milk production; as the number of nutrients consumed drives milk production [7,8]. Previous studies have also suggested possible associations between grazing time of a cow with its milk production level [9,10]. Thus, the knowledge obtained through studying grazing and rumination behaviours can be applied to effectively address animals’ demands for pasture or additional feeds and to improve animal welfare and productivity in a grazing-based system [11].

The time a cow spends grazing depends on its nutritional requirements and the type and availability of feed [12], and the time it spends ruminating depends on the quality of feed and additional supplements consumed [13,14]. The amount of time utilized for grazing and rumination activities may vary due to various elements related to the animal itself, the pasture, the environment, and the management [15,16,17]. In addition, the length of time spent ruminating is influenced by the time spent grazing, herbage intake, neutral detergent fibre content, and particle size of the forage [18,19]. In grazing animals, motivation for grazing is influenced by both internal (e.g., physiological and metabolic responses and stimulation for feed intake as a result of hunger) and external (such as sensory characteristics of food) drivers [20]. Positive sensory stimulations including taste, smell, and palatability trigger grazing motivation that mostly results in higher intake and subsequently higher milk yield [21,22]. The animal’s motivation to eat is further stimulated by the sight and sound of other nearby eating animals [23]. Thus, not only appetite or satiety but also hedonic and motivational factors linked with food also affect grazing behaviour [23]. In addition, inherent differences in grazing and rumination behaviours exist among cows differing in intake capacity and production efficiency [24]; high-producing cows generally require a longer period of grazing to fulfil their nutritional requirements. Likewise, varying milk production levels of dairy cows due to different lactation years and different stages (early, mid, late) within a lactation influence feed demand, and are expected to have effects on grazing and rumination times [25,26]. Young cows in the first year of lactation have different feed demands and hence different behaviour than mature cows in higher lactations. Variations in the quality of pasture and the type of pasture consumed during various seasons are additional factors impacting herbage intake [27,28,29], and therefore expected to influence grazing and rumination times [30]. Grazing dairy cows are fed supplements to address their energy and protein demands when quality pasture is less available which is also believed to be modulating their grazing time and rumination time [31].

New Zealand dairy herds are primarily populated with three breeds, Holstein-Friesian (HFR), Jersey (JE), and KiwiCross (KC, Crossbreed of Holstein-Friesian/Jersey). Cows rely mainly on grazing pasture as a major dietary component and receive additional supplementary feeds when the availability of quality pasture is compromised due to dry weather conditions. In a grazing system, animal behaviour is more unpredictable as animals have to adapt to constantly changing sward and weather conditions [32]. Therefore, behavioural variations are potentially difficult to identify in grazing animals. Moreover, in a grazing system, the average behaviour of the whole herd is expressed as an indicator of the external and/or internal drivers of each animal in the herd; this is because measuring individual animals’ behaviour has been a challenge in the past, whereas individual animals vary in expressing distinct and consistent behaviour [33]. Recent advances in sensor-based Precision Livestock Farming (PLF) tools offer opportunities to automatically monitor and record grazing and rumination behaviours of individual animals on a real-time basis [34,35,36]. Over the last decade, growing appeals for PLF devices have increased the number of studies describing the eating and ruminating behaviours of dairy cows [37,38]. However, studies focusing on New Zealand cows are limited in the literature to date. For example, a previous study explored differences in grazing and rumination behaviours of individual dairy cow breeds [39], but their study did not account for other factors including lactation year/parity of cow, season, and supplementary feeds consumed by animals.

Grant and Albright [40] concluded that management-related factors including grouping strategy, feeding system, quality of the feed consumed, as well as social hierarchy and competition for feed and water, all influenced the feeding behaviour of indoor cattle. However, these aspects remain unexplored in New Zealand’s grazing-based dairy system. For instance, how do cows differ in their grazing and ruminating behaviours when kept as a single herd in which animals of different breeds in different lactation years graze together? Do animals with varying breeding worth indexes vary in their grazing and ruminating behaviours? How do different days in milk over the lactation period affect grazing and rumination behaviours? How do seasonal variations and feeding supplements influence behaviours? What are the most substantial sources of variance in grazing and rumination behaviours? Furthermore, grazing time and the subsequent time required for rumination are partially interdependent and are limited or prolonged by each other. Thus, a longer grazing time may result in higher intake, hence may require a longer rumination time to process the ingested feed. However, a negative association might exist at some levels, as cows cannot graze and ruminate at the same time. In that scenario, is grazing time driving rumination time even if the cows are consuming additional supplementary feeds? A comprehensive understanding of these mechanistic connections is essential in a pasture-based system to develop strategies to optimize dairy cow production through better management of pasture and feed availability [41,42]. Understanding variation in the behaviours among cows of different breeds and lactation years, and consuming different supplementary feeds in different seasons should contribute to creating tailored management that better meets the needs of different animals on the farm. This information could also be helpful to select better and more profitable replacements.

The quantity of feed required to reach the satiety needs varies greatly and depends on age (or lactation year), state of production (days in milk) and breed [13]. Thus, we hypothesized that the times spent grazing and ruminating should vary among dairy cows of different breeds, breeding worth index, and lactation year due to varying levels of production (days in milk) and feed demands. We further hypothesized that grazing dairy cows change their grazing and rumination times in varying conditions related to different seasons that affect pasture quality [27], and also when animals are fed supplements [28]. As grazing partially drives rumination, we also, assumed that grazing time and rumination time are related, and the strength of this relationship varies depending on the breed and lactation year of the cow, and seasons. Therefore, the objectives of this study were to explore the variation in grazing and rumination times over the lactation period in grazing dairy cows, considering the effects of breed, lactation year, and their interaction while accounting for breeding worth index, days in milk, and supplementary feeds. This study also evaluated the relationship between grazing time and rumination time, and if the strength of the relationship varies as affected by breed, lactation year, and season/supplementary feedings. This study further evaluated the magnitude of variance in grazing and rumination times explained by different study factors and their interactions.

## 2. Materials and Methods

### 2.1. Grazing Conditions

The study was performed at Dairy Unit 1, Massey University, Palmerston North, New Zealand (Latitude: −41.3009, Longitude: 174.7720). Dairy unit 1 is a pasture-based, once-a-day milking dairy farm operated through a rotational grazing scheme with a spring calving system. The farm area consists of 142.7 hectares and is divided into 63 paddocks. The local climate is of a temperate type with four seasons classified as spring (September to November), summer (December to February), autumn (March to May), and winter (June to August). The annual average temperature in the area over the study period was ~16 °C (8–24 °C) with an annual rainfall of ~960 mm [43].

### 2.2. Grazing Animals

Spring-calved, lactating and pasture grazing dairy cows (n = 162) were used in the current study. Study cows were a subset of the whole herd (n = ~260) at the farm, they grazed together along with the other cows and altogether managed as one herd. The study period consisted of three years, a subgroup of 54 cows was randomly selected each year (54 × 3 = 162). The cows were not completely independent in the three study years because some of them were used more than once by random resampling within the available cows. The selection of cows was based on their breed affiliation, lactation year, and breeding worth (BW) index value. The cows selected each study year were of three breeds, Holstein-Friesian (HFR), Jersey (JE), and Holstein-Friesian/Jersey Crossbreed (KiwiCross) with 18 cows of each breed (18 × 3 = 54). Those 18 cows in each breed category were of 3 different lactation years (with 6 cows of each lactation year, 6 × 3 = 18). The cows were either in their 1st, 2nd, 3rd, or 4th lactation years. The 6 cows within each lactation year had different breeding worth index values (103 < BW > 151). The BW index value is the measure of the genetic merit of the animal for farm profit [44]. Breeding Worth (BW) is the index used to rank cows and bulls on their expected ability to breed profitable, efficient replacements. BW is calculated by combining breeding values (An estimate of a cow or bull’s genetic merit for a trait) with the appropriate economic values (An estimate of the future dollar value of a unit change in each trait) for each trait and adding them all together. The cows were altogether kept in the same grazing paddocks all the time throughout their lactation period (~270 days) except when brought to the milking shed in the morning at 05:00 a.m. Cows were rotated from one paddock to another based on the natural grass production and growth cycle.

### 2.3. Feeding of Experimental Animals

The feeding regimes of the cows over three study years are shown in Table 1. The feed requirements for the pasture grazing cows were established by the farm manager based on the feed requiement table by DairyNZ [45]. The cows mainly grazed pasture of perennial ryegrass (*Lolium perenne* L.) mixed with red clover (*Trifolium pretense*) and white clover (*Trifolium repens*). Besides pasture, cows grazed chicory (*Cichorium intybus*) as well in the spring season. To meet energy requirements and to cope with the seasonal changes in pasture quality and production [46], cows were additionally fed with supplements including maize (*Zea mays*) silage, corn gluten (*Zea mays* L.), tapioca (*Manihot esculenta*), turnips (*Brassica rapa subsp. rapa*), and distillers’ grains during summer and autumn seasons. Replacing good quality pasture with an alternative feed source or ‘balancing pasture’ is not considered advantageous; therefore, supplements are only used to provide energy when there is insufficient pasture available especially during summer and autumn. Supplementary feeds are used when quality pasture is less available, to fill the feed deficits and to support the cows to maintain energy intake and production [47]. Moreover, the purpose of providing supplements to milking cows in autumn is also to achieve calving body condition score (BCS) targets, if the feeds are not supplemented, cows are more prone to lose as quality pasture is insufficient at that time of the year. Distillers’ Grains (DG), corn gluten (CG), and tapioca were usually fed in the feeding area in the milking shed after milking, whereases, maize silage (MS), grass silage (GS), and turnips were fed around midday in the paddock. CG and DG were fed in the form of pellets, tapioca in the form of ground meal, and turnips stems, and leaves were fed in situ. The supplementary feeds were provided in equal amounts to all cows and were equally accessible to each cow. However, the actual intakes of either the grass or supplements were not measured. The cows had ad libitum access to drinking water in each paddock.

### 2.4. Behaviour Recording

An automated device, AfiCollar (Afimilk Ltd., Kibbutz Afikim, 1,514,800, Israel) was used to continuously monitor and record the time spent grazing and ruminating by the cows. The collar device was validated for measuring grazing and rumination behaviours in grazing dairy cows [48]. AfiCollar device monitored and recorded the minute-by-minute behaviour for consecutive 24 hours throughout the lactation period for three study years. The collar device had a triaxial (x, y, z) accelerometer-based sensor that was fitted within a box attached to the collar and positioned on the right side of the animal’s neck. The sensor could identify and classify specific behaviour categories such as grazing, and rumination based on the patterns of the animal’s head movements. The data collected by the sensor were analyzed by the collar device using built-in generic algorithms and produced as min/h behaviour counts (grazing time and rumination time). The data collected by the AfiCollar device were recorded and subsequently transmitted wirelessly to a base station through Wi-Fi while cows were in the range of ~500 meters. The data for the individual cows were manually downloaded in a Microsoft Excel spreadsheet (Version 2016, Microsoft corporation, Redmond, Washington, USA. Retrieved from https://office.microsoft.com/excel) from the computer attached to the base station, and separately sorted.

### 2.5. Data Collection and Preparation

Grazing time and rumination time of the individual cows were recorded over the lactation period for three consecutive years (2018 to 2021). The lactation period of the cows usually spanned between August to April of the next year (~270 days), following the typical New Zealand spring calving system. The lactation period covered spring, summer, and autumn seasons, while cows were at the dry stage in winter.Data were collected only when cows were at the milking stage, thus no data were collected in winter. Data collection for each cow in each study year started once the cow calved and ended when it was dried off. The lactation period for 2018–2019 was named Year-1, the lactation period for 2019–2020 was named Year-2, and the lactation period for 2020–2021 was named Year-3.

The frequencies of behaviour activities summarized by the AfiCollar device were minutes within an hour (min/h) utilized for grazing and rumination. The minutes per 24 h (min/day) spent grazing and rumination were manually calculated using the min/h data. Daily grazing time (min/day) and rumination time (min/day) of the individual cows along with their progressing days in milk (DIM, from the day of calving until the day of drying off) and BW index value were sorted separately for each year over the study period. The data collected were further classified into different breeds, lactation years, and seasons.

### 2.6. Data Analysis

#### 2.6.1. Variation in Grazing and Rumination Behaviours

A general linear mixed model fitted in a factorial design with breed × lactation year and their interaction while accounting for days in milk, breeding worth index, individual cow, season, feeding regime within the season, and their interactions was performed in SAS version 9.4 (SAS Institute Inc., Cary, NC, USA) to test the differences in grazing and rumination times. Grazing time and rumination time were the main dependent variables. Breed, lactation year, and their interaction were the main fixed effects, while individual cows nested within breed and lactation year was included as a random effect in the model. As lactation length ranged from August to April next year and covered three seasons (spring, summer, and autumn), the season was included in the model as a fixed factor to test its effect as well as its interaction with the other fixed effect on grazing and rumination times. Cows received different supplementary feeds during different seasons in each study year, so, the feeding regime nested within the season was added as a fixed effect in the model. The BW index value, one of the ranking variables for the study cows was also added as a covariate in the model. The calving date was different for each cow, therefore, DIM was added as a continuous covariate in the model. The interactions between the covariates (BW, DIM) and the fixed effects (breed, lactation year, and season) were also included in the model to test if the relationship between covariates and grazing/rumination varied between the fixed effects.

To further determine the relative effect size or the strength of various study factors and their interactions, variance partitioning was used considering the type I sum of squares values of the study factors and their interactions. The significance and effect size of the study factors were assessed separately for each study year because the study years differed in supplementary feeds as well as the lactation year of the study cows. The model used in this study is given below:

Y_ijklmn_ = μ + A_i_ + B_j_ + A_i_ × B_j_ + *C*_k_ (A_i_ × B_j_) + D_l_ + E_m_ (D_l_) + A_i_ × D_l_ + B_j_ × D_l_ + A_i_ × B_j_ × D_l_ + X_i_ + Y_j_ + X_i_ x A_i_ + Y_j_ × A_i_ + X_i_ × B_j_ + Y_j_ × B_j_ + X_i_ × A_i_ x B_j_ + Y_j_ × A_i_ × B_j_ + X_i_ × D_l_ + Y_j_ × D_l_

where: Y_ijklmn_ is the k^th^ observation in the i^th^ treatment group A and j^th^ treatment group B and so on; μ is a general mean; A_i_, B_j_ represent the fixed effects of breed and lactation year; A_i_ × B_j_ represent interaction between breed and lactation year; C_k_ (A_i_ × B_j_) is random effect of cow within breed and lactation year; D_l_ is fixed effect of season; E_m_ (D_l_) is supplementary feed within season; A_i_ × D_l_ is interaction between breed and season; B_j_ × D_l_ is interaction between lactation year and season; A_i_ × B_j_ × D_l_ is interaction between breed, lactation year and season; X_i_ + Y_j_ are covariates as BW and DIM; X_i_ × A_i_ is interaction between BW and breed; Y_j_ × A_i_ is interaction between DIM and breed; X_i_ × B_j_ is interaction between BW and lactation year; Y_j_ × B_j_ is interaction between DIM and lactation year; X_i_ × A_i_ × B_j_ is interaction between BW, breed and lactation year; Y_j_ × A_i_ × B_j_ is interaction between DIM, breed and lactation year; X_i_ × D_l_ is interaction between BW and season; Y_j_ × D_l_ is interaction between DIM and season.

#### 2.6.2. Relationship between Grazing and Rumination Behaviours

The relationship between grazing time and rumination time and the possible differences in the regression lines between grazing and rumination times for different fixed effects were investigated with the same model. Grazing time was added as a covariateand rumination time was included as a dependent variable in the model. The interactions of grazing time with the breed, lactation year, and season were included in the model to test their significance for rumination time. Variance partitioning was used considering the type I sum of squares values to determine the effect size of the grazing time itself and its interactions with the breed, lactation year, and season on rumination time.

## 3. Results 

### 3.1. Variation in Grazing Behaviour

**Figure 1:**
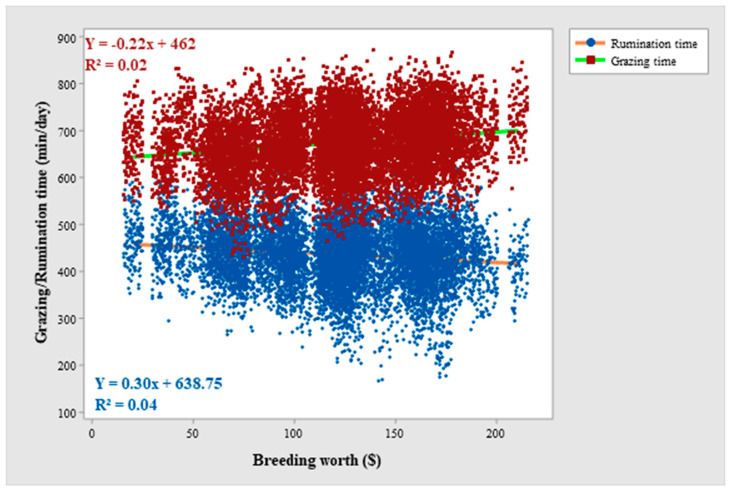
Scatterplot of grazing time (min/day) and rumination time (min/day) with breeding worth index value ($) of grazing dairy cows across the lactation period for Year-1 of the study period.

**Figure 2:**
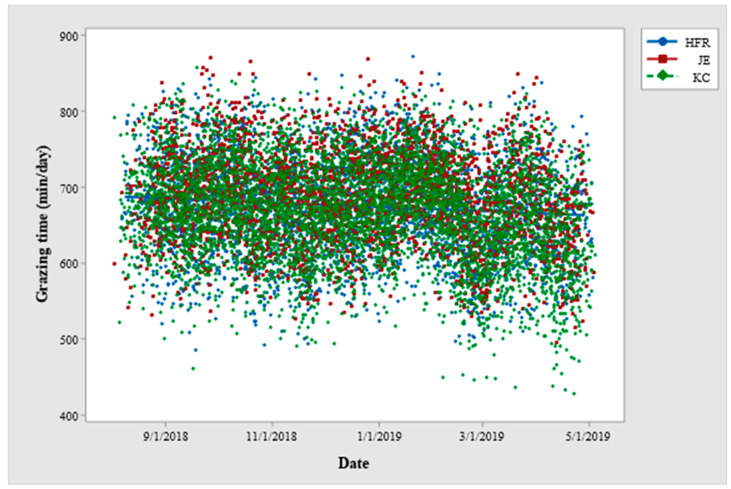
Scatterplot between date and grazing time (min/day) for Holstein-Friesian (HFR), Jersey (JE) and KiwiCross (KC) cows across the lactation period for Year-1 of the study period. (The scatterplot is based on the raw values of grazing time).

**Figure 3:**
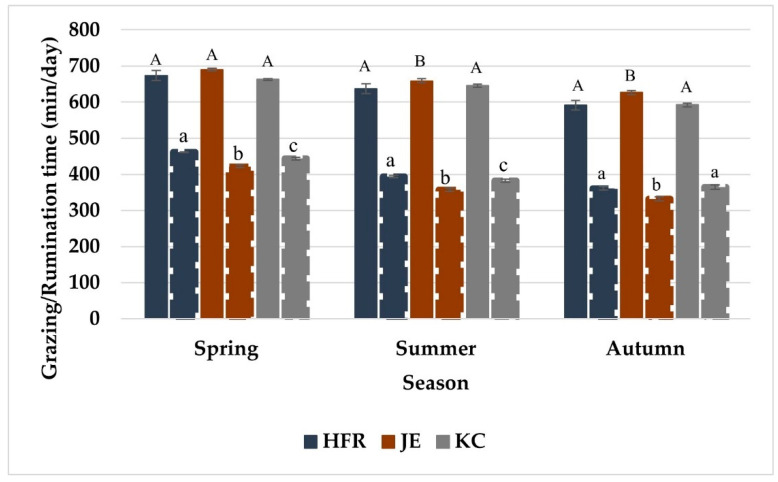
Grazing time (min/day) and rumination time (min/day) of Holstein-Friesian (HFR), Jersey (JE) and KiwiCross (KC) cows across three seasons over the lactation period for Year-1 of the study period. Compact bars show grazing time and dotted bars show rumination time. Error bars represent standard error. Letters on each bar show significant differences between bars, capital letters for grazing time and small letters for rumination time.

**Figure 4:**
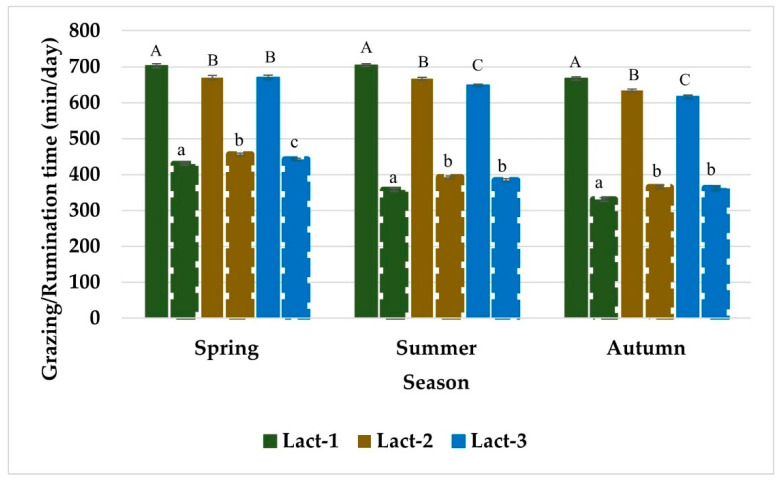
Grazing time (min/day) and rumination time (min/day) of dairy cows in lactation year 1 (lact-1), lactation year 2 (lact-2) and lactation year 3 (lact-3) across three seasons over the lactation period for Year-1. Compact bars show grazing time and dotted bars show rumination time. Error bars represent standard error. Letters on each bar show significant differences between bars, capital letters for grazing time and small letters for rumination time.

Grazing time differed among the individual cows within breed and lactation year throughout the study period (Table 2). Grazing time varied among different seasons and the feeding regimes within each season throughout the study period. The daily time spent grazing was longest in spring, it was reduced in summer, and reached a minimum in autumn. Grazing time was affected by the breeding worth (BW) index and days in milk (DIM) of the cows (Figure 1 and Figure 2). Grazing time varied among different breeds in Year-2 and Year-3, although, the breed effect was not highly significant in Year-2 (*p =* 0.037) and not significant in Year-1 of the study period. Jersey (JE) cows grazed longest followed by Holstein-Friesian (HFR), and KiwiCross (KC) among the three breeds. Grazing time varied among cows depending on their lactation year in Year-1 but not in Year-2 and Year-3 of the study period with a decrease grazing time in the cows in higher lactation years. Breed and lactation year showed no statistical interaction for grazing time. Breed (except Year-3) and lactation year (all study years) had interactions with the season (Figure 3 and Figure 4), and the trend of a gradual decrease in grazing time from spring to autumn was evident for each breed and each lactation year. Jersey cows among the breeds and first lactation cows remained the longest grazers in spring, summer, and autumn. The effect of BW depended on the breed in Year-1 and Year-2, while BW and lactation year never jointly influenced grazing time. DIM had statistical interaction with lactation year in Year-1 and Year-2 of the study period, while DIM never showed interaction with breed.

The analysis further showed that the total amount of variance in grazing time explained by the study factors and their interactions included in the model in Year-1, Year-2, and Year-3 of the study period was 35.60%, 39.42%, and 39.23%, respectively (Table 2). The effects of individual cows (Year-1 = 16.77%, Year-2 = 20.6%, Year-3 = 12.36%), season (Year-1 = 5.60%, Year-2 = 8.59%, Year-3 = 12.18%), and feed (Year-1 = 2.50%, Year-2 = 3.67%, Year-3 = 6.30%) consistently remained the factors explaining most of the variance in grazing time. Breed accounted for 1.71%, 3.80%, and 5.76% of the variance in grazing time in Year-1, Year-2, and Year-3, respectively. The lactation year of the cows in Year-1 explained 6.3% of the variance in grazing time and 1.75% and 1.23% in Year-2 and Year-3 of the study period, respectively. The interaction of breed and lactation year did not explain much of the variance (1.5%) and both BW and DIM described <1% of the variance in grazing time. The statistical interactions of different factors included in the study design explained very low amount of variance (<1%) in grazing time.

### 3.2. Variation in Rumination Behaviour

**Figure 5:**
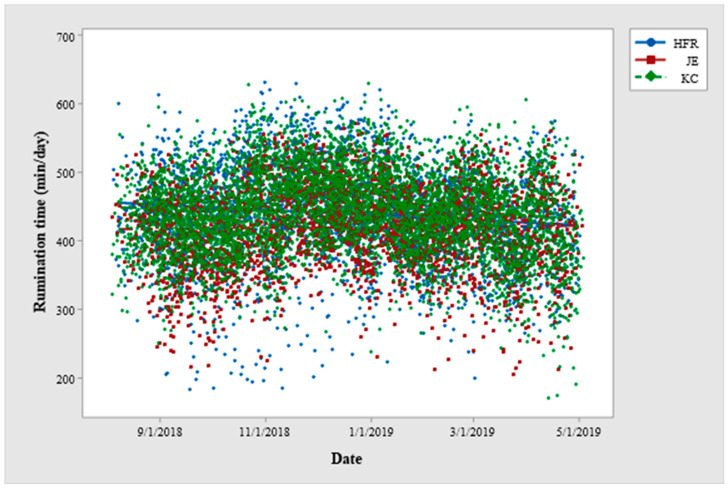
Scatterplot between date and rumination time (min/day) for Holstein-Friesian (HFR), Jersey (JE) and KiwiCross (KC) cows across the lactation period for Year-1 of the study period. (The scatterplot is based on the raw values of rumination time).

Rumination time varied among the individual cows within breed and lactation year throughout the study period (Table 3). Rumination time was different for seasons and for varying feeding regimes within each season in all study years. Rumination time was affected by DIM in all study years while the effect of BW was only observed in Year-2 and Year-3 of the study period (Figure 1 and Figure 5). Rumination time did not vary among breeds (except for Year-3) and it was shortest for JE and longest for HFR throughout the study period. Rumination time never differed in cows in different lactation years. Breed and lactation year showed no statistical interaction for rumination time, while both breed (except Year-3) and lactation year (all study years) had interactions with the season (Figure 2 and Figure 3). The overall trend of a gradual decrease in rumination time from spring to autumn was evident for each breed and lactation year. Regardless of the season HFR remained the longest ruminator among the breeds, and the cows in the first year of lactation remained the shortest ruminators. BW showed interaction with the breed in Year-1 and Year-3, and with lactation year in all years of the study period. DIM had an interaction with breed and lactation year throughout the study period.

The analysis further showed that the study factors tested in the model and their interactions explained 40.02%, 41.05%, and 40.02% of the total variance in rumination time in Year-1, Year-2, and Year-3 of the study period, respectively (Table 3). Individual cow (Year-1 = 24.03%, Year-2 = 14.54%, Year-3 = 16.07%), season (Year-1 = 2.31%, Year-2 = 16.98%, Year-3 = 7.41%), and feeding regime within the season (Year-1 = 1.70%, Year-2 = 2.11%, Year-3 = 1.64%) explained the maximum amount of variance in rumination time. BW explained 0.71%, 0.79%, and 0.81%, while DIM described 0.12%, 0.05%, and 0.36% of the variance in Year-1, Year-2, and Year-3, respectively. Breed effect accounted for 2.19%, 1.78%, and 8.54% while lactation year accounted for 1.77%, 0.05%, and 0.98% of the variance in Year-1, Year-2, and Year-3 of the study period, respectively. The statistical interactions of different factors included in the study design explained very low amount of variance (<1%) in rumination time.

### 3.3. Variation in the Relationship between Grazing and Rumination times

**Figure 6:**
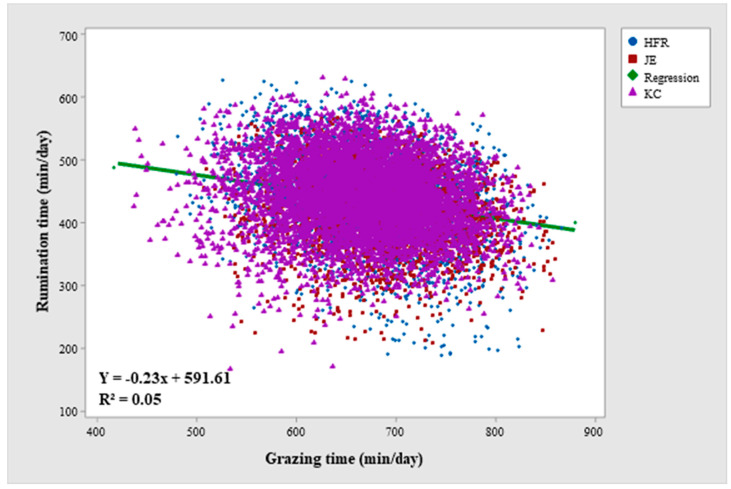
Scatterplot between grazing time (min/day) and rumination time (min/day) for Holstein-Friesian (HFR), Jersey (JE) and KiwiCross (KC) cows across the lactation period in Year-1 of the study period. (The scatterplot is based on the raw values of grazing time and rumination time).

Grazing time overall affected rumination time throughout the study period (Table 4), and there was a negative relationship between both variables (Figure 6). The regression line between grazing time and rumination time among breeds remained the same, while it differed among lactation years in only Year-1 of the study period. The regression line between grazing and rumination varied for different seasons in Year-1 and Year-3 of the study period. The statistical interactions of grazing time with BW (except in Year-2) and DIM (except in Year-1) for rumination time were not significant.

The total amount of variance in rumination time explained by grazing time as a covariate in the model was 5.14%, 2.17%, and 2.14% in Year-1, Year-2, and Year-3 of the study period, respectively (Table 4). In other words, the addition of grazing time in the model improved the variance explained in rumination time, i.e., for Year-1 from 38.02 to 43.76; for Year-2, from 41.5 to 44.35; and for Year-3, 39.5 to 42.4. The amount of variance in rumination time explained by the interaction of grazing time with breed, lactation year, season, BW and DIM remained very low (<1%) throughout the study period.

## 4. Discussion

The current study evaluated the variation in grazing time and rumination time and their relationship as affected by breed, lactation year, breeding worth (BW) index, and days in milk (DIM) of the individual cow, season, supplementary feeding, and their interactions. In the current study, the cows were managed altogether with other (non-study) cows as a single herd, like normally on farms in New Zealand. Thus, interactions among the cows within different breed or lactation year,, and effect by herd size cannot be excluded but were not the focus of this study. Furthermore, the feeding regimes (i.e., herbage from pasture and supplementary feeds) differed among the study years, similar to the lactation year of the cows, this may explain variation in the results among different years (Year-1, Year-2, Year-3) of the study period, and this was the reason for analyzing the dataset separately for each study year.

Grazing and rumination times varied among the individual cows in all years of the study period. The analysis further indicated that individual cows within breed and lactation years have been the main contributors to the variance in grazing time (12–20%) and rumination time (14–24%). This might be due to the variability in grazing and digestive efficiencies, genetic potential or individual traits of each study cow [39]. Variation in the individuals’ behaviour describes variation in their personality traits [49]. For example, animals that are under highly competitive pressure may exhibit different ingestion behaviours to those with less competition. The foraging behaviour of grazing animals is not a simple process, but rather an outcome of interactions between feed and the animal itself [50]. Several animal-related factors influence their diet selection, bite mass and bite rate [51], such as the mechanism of harvesting food; cows mainly use their tongue to harvest forage and they have a large mouth for a large bite. Animals tend to choose a diet of higher quality; for example, they select clover over grass and leaves over the stem and dead material [52]. Moreover, grazing management practices, the state of the grazed pasture, pasture availability and the quality or composition of pasture have significant effects on the selection of herbage by the animal [53,54]. Furthermore, previous experience (e.g., previous feeding regime), the physiological status of the animals, and the digestive processes also influence their drive for grazing and choice of pasture [55]. These findings emphasize that it is crucial to consider individual dairy cows when making management decisions. This study considered a few animal-related factors such as breed, lactation year, and breeding worth. Some other characters such as grazing efficacy, nonvisual traits, and social status of the individual animal in herd were not studied, and can be further explored in terms of their effects on grazing and rumination behaviours.

The season greatly influenced grazing time of dairy cows and explained 5–12% of the variance in grazing time. The cows used in the current study calved in spring following the normal calving pattern in New Zealand dairy system. Grazing time tended to increase during the initial weeks of lactation period in spring, reduced in summer, and further declined towards the end of lactation in autumn. These findings were consistent with a recent study that found a significant effect of season on grazing time in lactating dairy cows [56]. Few other studies also observed a significant effect of season on grazing time and comparatively longer grazing time at the initial weeks of lactation in spring than that in summer or autumn [38,57]. It has been reported that both milk yield and herbage intake (indicated by grazing time) increase during the first few weeks of lactation, and gradually decrease towards the end of lactation [58]. Furthermore, both herbage intake and time spent grazing in dairy cows increase during the early lactation and decline towards the end of lactation, going parallel with the milk production curve [59]. The decline in grazing time in summer in the current study could be potentially due to the high-temperature humidity index that could have induced heat stress and resulted in reduced grazing time [60]. Additionally, dry summer affects the pasture quality and leaves the grass mostly rich in fibre content. Reports state, that when fed a moderate to a high-fibre diet, cows avoid consuming long particles and decrease their eating time per meal, but usually increase the number of meals per day [61]. Thus, the shorter grazing time in the cows in summer was most probably due to heat stress in cows and drought effect on pasture. Moreover, cows were fed additional supplements to address their nutritional demands during summer which would also have caused a reduction in grazing time. Grazing time gradually decreased to a further level in autumn. The even shorter grazing time during autumn was because the cows were at the end of the lactation period and were going to be dried off. Additionally, their nutritional demands were addressed by the additional supplementary feeds mainly during summer and autumn as these seasons are dry and potentially influence pasture quality (low ME and a high proportion of dead tissue) and availability in New Zealand [62]. The cows had lower feed demands in autumn and thus spent less time grazing. It has also been reported that grazing behaviour such as sward selection, time spent grazing, and consumption rate is affected by pasture management, type and quality of pasture, and supplementation [63]. The difference in grazing time was not large when cows consumed chicory, silage, or sole pasture. Whereas there was a reduction in grazing time (~60 min/day) when supplements were fed to the cows in autumn. The same effect, a reduction in the grazing time of 8.5 min per cow per day (overall 63 min per cow per day) for each kilogram of supplement consumed has been reported [64]. Another study reported 54 min reduction in the grazing time when fed with 14 kg of supplement per cow per day [65]. Thus, the overall pattern and length of grazing time in different seasons and for different feeds found in the current study are consistent with the previous reports. However, the supplementary feeds were provided to all the animals at the same time so there is a probability that some animals might have consumed more or less than the required and this might have resultantly affected their grazing and rumination times. These findings inform the varying requirement of pasture and additional feeds over the lactation period by grazing dairy cows, and could be considered for making feed management decisions fr grazing cows

Season significantly affected rumination time and explained 2–17% of the variance in it in the current study. The effect of season was potentially linked to the supplementary feeds provided to the cows within different seasons as the type and quality of diet affect rumination time [17]. The time spent rumination directly depends on the time spent eating, and the feed quality and type. Therefore, the effect of season on the variation in grazing time was further reflected in the variation in rumination time as well. Moreover, rumination time showed a seasonal curve parallel with the grazing time. These findings were consistent with a previous study [56] that reported a similar trend in rumination time during different seasons except that rumination time remained increasing until the end of summer. Whereas in the current study rumination time declined in summer, which was potentially due to heat stress caused by the high temperature humidity index [66]. The seasonal effect on variation in rumination time was also related to the varying feeding regimes in each season. Rumination time was lowest when supplementary feed was included in the daily ration in autumn. This was probably due to the low particle size and less nutrient-detergent fibre content of the supplementary feed which are principal drivers of rumination time [67].

Grazing time differed among the breeds with the highest significance in Year-3 of the study period. The magnitude of variance in grazing time described by the breed effect was accordingly highest in Year-3 (5.76%). Grazing time tended to be longer for Jersey (JE) cows and shorter for KiwiCross (KC) cows than that for HFR cows. Grazing time among Jersey, Holstein-Friesian (HFR), and Crossbreed cows have been compared in a few previous studies with varying results. Similar grazing times among HFR, Crossbreed, and JE cows (646, 637, and 662 min/day) have been reported with a comparatively longer grazing time than HFR (171 vs. 129 min) when expressed as per 100 kg body weight [39]. Similar findings have been reported by a recent study focuding on the effect of breed on eating time of cows kept in the indoor system [38]. Higher daily eating time by JE (382 min) compared to that of HFR (360 min) in total mixed ration-fed lactating cows have been previously observed [68]. Furthermore, studies reporting significant differences in time spent grazing among different breeds with longer eating time by JE cows are also available [69]. the bite mass is influenced by the constraints due to the anatomy including both mouth and body size of the animal [70]. Therefore, higher grazing time in JE cows was probably due to their smaller physical size (short body and small stature) that only supported a smaller bite mass, and it took JE cows a longer time to fulfill their satiety needs. Additionally, lower bite mass and grass intake by JE cows compared to HFR cows have been reported [39] which further justifies their grazing time to be longer. Thus, the difference in time spent grazing by cows of different breeds in the current study agrees with previous reports.

Rumination time was influenced by breed in only Year-3 of the study period with 8.53% of the variance in rumination time explained by the breed effect. The significance of the breed effect was relatively higher for both grazing and rumination in Year-3 which might be linked to consumption of different supplementary feeds and chewing behaviour or herbage intake with different nutritional demands for that study year. Significant differences in rumination time between Holstein-Friesian (10.4 h/day) and Jersey cows (9.0 h/day) with a similar quantity of intake have been reported; Holstein-Friesian cows spent more time (1.4 h/day) ruminating [68]. Rumination time remained longest for HFR cows and shortest for JE cows in the current study. This was consistent with previous findings that reported lower rumination time in JE than that in HFR [18,39,68]. Smaller-sized JE cows have been observed to have smaller bolus sizes due to the anatomical influence on bolus movement during rumination [39]. The study further suggested that inherent grazing and ruminating differences do exist between cows varying in intake capacity and production efficiency. Thus, the longer rumination time in HFR cows compared to JE cows can be explained by anatomical differences in the muzzle and incisor breadth between both breeds [70].

Grazing time did not vary among cows in different lactation years except in Year-1 of the study period, when first-lactation cows were included as study animals. Moreover, lactation year explained the highest (6.3%) amount of the variance in grazing time in Year-1 compared to Year-2 (1.8%) and Year-3 (1.2%) when study cows were in their 2nd, 3rd, and 4th years of lactation, and the effect of lactation year was non-significant. Eating time in dairy cows was not influenced by age or lactation year in a previous study; However, their study reported more variation in the eating time of heifers than that of mature cows [71]. In addition, we found a decreasing trend in grazing time with an increase in the year of lactation; the first-lactation cows showed the longest grazing time followed by the cows in their second, third, and fourth lactations. A decline in eating time and the number of chews with advancing age in dairy cows has been reported [71]. Shorter chewing times per unit of feed-in multiparous cows compared with primiparous cows have also been previously observed [72]. First-lactation or young cows show different grazing behaviour than mature cows (cows in 2nd, 3rd, or 4th lactation years), as young cows have a smaller body size, take smaller bites, and eat more slowly, hence spending a long time eating compared [73]. The first-lactation cows are still in the growing phase and need additional energy and protein to support growth and maintenance requirements. Their study further concluded that mature cows are socially more dominant, therefore, when housed together, younger cows eat (10 to15%), and rest (20%) less than when housed separately [74,75]. In the current study, all the experimental animals from different breeds and in different lactation years were grazed together as a single herd in the same paddock. The smaller bites due to smaller size, additional growth demands and being socially influenced by mature cows in the herd were presumably the potential reasons for the longer grazing time in first-lactation cows. Social status and nutritional requirements of young cows was not focus of this study, but it can be explored in the upcoming research.

The lactation year of cows never influenced rumination time during the study period and explained up to 1.77% of the variance in rumination time. Although grazing time varied between first-calvers and mature cows in Year-1, the rumination time was similar between young and mature cows in the subsequent lactation years (2nd, 3rd, 4th). These findings need further exploration in terms of comparisons of grazing and digestive efficiencies between first-lactation cows and mature cows. Moreover, rumination time declined in cows with an increase in lactation year which might be due to the increased digestive efficiency of mature or multiparous cows for fibrous feed, this needs further exploration. The results were opposite to previous studies which found an increase in rumination time in multiparous cows [76,77], and with no parity effect. The animals in those studies were kept indoors and fed on a concentrated diet. This was probably the main difference between those studies and the current study for contrasting trends of rumination time (pasture vs. total mixed ration), although the specific lactation years of the multiparous cows were not mentioned.

The lactation year and breed of the cows never showed statistical interaction with each other for grazing time or rumination time. Even though first lactation cows differed in grazing time (in Year-1), the difference was not breed-dependent. The findings in this study suggest that cows in early lactation require a comparatively longer time to graze and a larger allocation of pasture and/or supplementary feeds to address their satiety needs when they are grazing together with mature cows in a mixed herd. These findings should further help to manage the pasture and additional feed supply for a grazing herd with cows in different lactation years. Both breed and lactation years showed statistical interactions with the season for grazing time, and that was probably related to the varying supplementary feeds in each season. Studies have reported the effects of season and diet on variation in grazing time both indoor and outdoor dairy production systems [29,64]. Irrespective of breed affiliation or year of lactation, cows showed a gradual reduction in grazing time from spring to autumn (from the start of lactation towards the end of lactation). These findings on the other hand reflect the trend in feed/pasture demand by dairy cows in different seasons over the lactation period. This information could be helpful to improve pasture management and utilization and additional feed supply on the farm effectively addressing the variable forage demand by the grazing cows in different seasons over the lactation period. This could be helpful to further improve the animal productivity; consistent allocation of sufficient pasture daily can lead to approximately 10% increased milk yield [78]. Thus, an accurate indicator of pasture availability and the appropriate time to deliver additional feedstuff would be a potential feed management tool, particularly in a grass-based dairy system.

Days in milk and breeding worth index of the cows had effects on their grazing time and rumination time but the amount of the variance in grazing time and rumination time explained by BW (0.03–0.2%, 0.7–0.8%) and DIM (0.03–0.5%, 0.1–0.4%) was very low. The significant effect of BW and DIM might be due to some unknown reasons whereas, DIM and BW originally did not affect grazing and rumination. This was further verified by the magnitudes of the effect sizes of BW and DIM which were very low for both grazing and rumination. Grazing and ruminating times were similar in high BW ($146) and low BW ($40) indexed animals in a recent study [79]. Another study [80] reported similar grazing times but a greater herbage intake rate for New Zealand dairy cows with modern genotypes (the 1990s) compared with those of an old genotype (the 1970s).

Grazing activity, to some extent, drives rumination activity, therefore, one of the objectives of the current study was to investigate the relationship between grazing time and rumination time and if this relationship varies for different breeds, lactation years, and seasons when animals are provided supplementary feeds. Grazing time and rumination time were significantly negatively correlated which is quite reasonable as when animals spend more time grazing, there is less time available for rumination. Longer periods of feed deprivation in grazing cows result in longer grazing bouts with higher intake along with a reduction in time left for rumination [81]. Grazing time interacted with lactation year for rumination time only in Year-1 of the study period. The inclusion of primiparous cows in Year-1 of the study period and their different grazing times compared to mature cows explains this interaction. Whereas there was no statistical interaction between grazing time and breed for rumination time in Year-1. Furthermore, this joint effect of grazing time with lactation year on rumination time only in Year-1 of the study was probably because lactation year also affected grazing time in Year-1 of the current study (Table 2). The interaction of grazing time with season could be explained by the varying supplementary feeding within each season. Along with other study factors, grazing time explained 5.54%, 2.17%, and 2.14% of the additional variance in rumination time in Year-1, Year-2, and Year-3 of the study period, respectively. This further means that although rumination time is influenced by grazing time, the variation in rumination time is not solely explained by grazing time. Some other factors including feed efficiency, type of feed offered, quality of feed, and time of the supplement offered might be affecting rumination time. This needs further exploration.

## 5. Conclusions

The current study to our knowledge is the first study that provides insight into variation in grazing and rumination times and their relationship in grazing dairy cows considering the combined effects of breed, lactation year, individual cow, season, supplementary feeds, breeding worth index, and days in milk. The individual cow had the largest effect on variation in grazing and rumination behaviours. Minor differences existed between Jersey and Holstein-Friesian cows in grazing and rumination times with JE being the longest grazers and HFR being the longest ruminators. The length of time spent grazing and rumination gradually decreased in cows with an increase in the year of lactation, which indicates cows in their first lactation need more time to graze to address their satiety needs. Grazing time and rumination time increased at the start of lactation in spring and declined towards the end of lactation in autumn. Additionally, supplementary feeds greatly affected grazing and rumination times in a way that cows substantially reduced their time spent grazing and ruminating when additionally offered supplements. Although rumination has a relationship with grazing, the variation in rumination time is not solely explained by grazing time. With all the factors considered (individual cow, breed, lactation year, season, supplementary feed, and their interactions) in the current study, we could only explain 35 to 39% and 40 to41% of the variance in grazing time and rumination time, respectively, and 60–65% of the variance remained unexplained. Individual cows, season, and supplementary feeds were the factors explaining most of the variance in grazing and rumination behaviours.

Due to large variation in grazing and rumi8nation behaviours among the individual cows, management decisions based on the individual animal in the herd are crucial, and are expected to support improvement in animal productivity and welfare leading to farm profitability. Our findings further indicate how pasture utilization and additional feeds can be adjusted over the lactation period, depending on the nutritional demands of dairy cows of different breeds and lactation years to improve their health, welfare, and productivity. Thus, an accurate indicator of pasture availability and the appropriate time to deliver additional feedstuff would be a potential tool, particularly in a pasture-based daiey system in New Zealand. Including digestive, metabolic, and social behavioural parameters holds great potential to learn more about what further influences grazing and rumination behaviours. As there were no uniform control herds, there are still some questions that the study design did not allow testing. Furthermore, highlighted areas including additional grazing components (e.g., intake rate, bite mass, jaw movements, feed efficiency, social status, and other behavioural traits) and testing pasture quality are potential opportunities for future studies.

## Figures and Tables

**Table 1:** Seasonal feeding regimes for grazing dairy cows during the study period.

Season	Year-1	Year-2	Year-3
Spring	Pasture, Chicory	Pasture, Chicory	Pasture, Chicory
Summer	Pasture, Turnips, GS	Pasture, Turnips, DG, Tapioca	Pasture, Turnips, GS
Autumn	Pasture, GS	Pasture, MS, DG, Tapioca	Pasture, MS, DG, Tapioca, CG

(Year-1, Year-2, and Year-3 represent the lactation period between 2018–2019, 2019–2020, and 2020–2021, respectively. GS = Grass silage, DG = Distillers’ grain, MS = Maize silage, CG = Corn gluten).

**Table 2:** Least square means (LSMean) and standard errors of means (SEM) of grazing time (min/day) for the effects of breed, lactation year and season, and *p* values and the variance (%) explained by breed, lactation year, cow within breed and lactation year, season, feed within the season, breeding worth (BW) index, days in milk (DIM), and their interactions in three consecutive years of the study period using a mixed effects model with the cow (*n = 54*) as a random factor, and BW and DIMas continuous covariates.

Grazing Time (Min/Day)						
Effect	Year-1		Year-2		Year-3	
Breed	LSMean	SEM	LSMean	SEM	LSMean	SEM
HFR	667	4.1	634^a^	3.3	632^a^	6.3
JE	669	3.7	658^b^	4.1	668^b^	6.4
KC	658	1.8	634^a^	1.8	629^a^	6.4
**Lactation year**						
1st	692^a^	3.6	-	-	-	-
2nd	657^b^	3.0	663	2.9	652	7.9
3rd	645^b^	3.4	637	5.8	635	8.5
4th	-	-	627	2.5	628	4.7
**Season**						
Spring	681^a^	2.0	675^a^	4.9	651^a^	6.5
Summer	673^b^	2.9	647^b^	5.2	666^b^	6.3
Autumn	639^c^	3.8	604^c^	5.0	598^c^	6.8
	**P**	**Var.**	**P**	**Var.**	**P**	**Var.**
Breed	0.1298	1.71	0.037	3.80	0.0002	5.76
lactation	0.0012	6.34	0.2034	1.75	0.1135	1.23
Cow (Breed*Lactation)	<0.0001	16.77	<0.0001	20.06	<0.0001	12.36
Season	<0.0001	5.60	<0.0001	8.59	<0.0001	12.18
Feed (Season)	<0.0001	2.50	<0.0001	3.67	<0.0001	6.30
Breed*Lactation	0.4443	1.52	0.9933	0.12	0.9243	0.24
Breed*Season	0.0002	0.13	<0.0001	0.22	0.0925	0.04
Lactation*Season	<0.0001	0.24	0.0243	0.07	<0.0001	0.13
Breed*Lactation*Season	0.0054	0.13	0.3072	0.06	<0.0001	0.17
Breeding worth (BW)	0.0346	0.03	<0.0001	0.18	0.0157	0.03
Days in milk (DIM)	0.0215	0.03	<0.0001	0.24	<0.0001	0.47
BW*Breed	0.0009	0.08	0.0009	0.09	0.4885	0.01
DIM*Breed	0.1658	0.02	0.9926	0.00	0.5631	0.01
BW*Lactation	0.6196	0.01	0.1694	0.02	0.1402	0.02
DIM*Lactation	0.0002	0.10	0.0099	0.06	0.2067	0.02
BW*Breed*Lactation	<0.0001	0.20	0.0107	0.09	0.0301	0.05
DIM*Breed*Lactation	0.0319	0.06	<0.0001	0.19	0.0874	0.04
BW*Season	0.0003	0.09	0.0791	0.03	0.0087	0.05
DIM*Season	0.0025	0.07	<0.0001	0.16	<0.0001	0.20
Total variance (%)	-	35.6	-	39.42	-	39.28

(Note: LSMeans that do not share a common letter are significantly different for the significance level set at the *p*-value of 0.05. HFR = Holstein-Friesian, JE = Jersey, KC = KiwiCross. P represents the *p*-value for the level of significance and Var. represents the variance in grazing time explained by the individual effects and their interactions included in the model. * indicates an interaction between study factors).

**Table 3:** Least square means (LSMeans) and standard errors of means (SEM) of rumination time (min/day) for the effects of breed, lactation year and season, and P values and the variance (%) explained by breed, lactation year, cow within breed and lactation year, season, feed within the season, breeding worth (BW) index, days in milk (DIM), and their interactions in three consecutive years of the stiudy period using a mixed effects model with the cow (*n = 54*) as a random factor, and BW and DIM as continuous covariates.

Rumination Time (Min/Day)						
Effect	Year-1		Year-2		Year-3	
Breed	LSMean	SEM	LSMean	SEM	LSMean	SEM
HFR	405	4.0	373	5.6	441^a^	5.6
JE	370	3.6	334	4.8	372^b^	2.9
KC	397	1.7	367	2.2	377^b^	4.9
**Lactation year**						
1st	372	3.5	-	-	-	-
2nd	404	2.9	371	3.4	381	8.8
3rd	396	3.3	390	6.8	375	9.5
4th	-	-	313	4.7	434	6.3
**Season**						
Spring	442^a^	2.0	417^a^	5.7	469^a^	7.2
Summer	378^b^	2.9	385^b^	6.1	388^b^	7.0
Autumn	353^c^	3.7	272^c^	5.9	333^c^	7.6
	**P**	**Var.**	**P**	**Var.**	**P**	**Var.**
Breed	0.1605	2.19	0.1116	1.78	<0.0001	8.54
Lactation	0.2256	1.77	0.936	0.05	0.258	0.98
Cow (Breed*Lactation)	<0.0001	24.03	<0.0001	14.54	<0.0001	16.07
Season	<0.0001	2.31	<0.0001	16.98	<0.0001	7.41
Feed (Season)	<0.0001	1.7	<0.0001	2.11	<0.0001	1.64
Breed*Lactation	0.8699	0.21	0.725	0.02	0.6768	0.05
Breed*Season	0.0002	0.23	0.0839	0.08	<0.0001	0.62
Lactation*Season	<0.0001	0.23	0.422	2.57	0.0413	0.01
Breed*Lactation*Season	<0.0001	3.5	0.1377	1.49	<0.0001	2.54
Breeding worth (BW)	<0.0001	0.71	<0.0001	0.79	0.0966	0.81
Das in milk (DIM)	<0.0001	0.12	<0.0001	0.05	<0.0001	0.36
BW*Breed	0.0021	0.07	0.305	0.02	0.0209	0.04
DIM*Breed	0.0028	0.07	<0.0001	0.27	<0.0001	0.1
BW*Lactation	0.0007	0.08	<0.0001	0.14	0.0004	0.08
DIM*Lactation	<0.0001	0.52	0.0035	0.07	0.0027	0.06
BW*Breed*Lactation	0.1997	0.03	<0.0001	0.35	<0.0001	0.12
DIM*Breed*Lactation	<0.0001	0.22	<0.0001	0.15	0.0244	0.05
BW*Season	0.0022	0.07	0.0233	0.05	0.8963	0.01
DIM*Season	<0.0001	2.04	<0.0001	1.51	<0.0001	0.52
Total variance (%)	-	40.02	-	43.01	-	40.0

(Note: LSMeans that do not share a common letter are significantly different for the significance level set at the *p*-value of 0.05. HFR = Holstein-Friesian, JE = Jersey, KC = KiwiCross. P represents the *p*-value for the level of significance and Var. represents the variance in grazing time explained by the individual effects and their interactions included in the model. * indicates an interaction between study factors).

**Table 4:** P values and the variance explained for the linear relationship between grazing time and rumination time while accounting for the effects of breed, lactation year, cow within breed and lactation year, season, supplementary feeds within the season, breeding worth (BW) index, days in milk (DIM), and their interactions using a mixed-effects model with the cow (*n = 54*) as a random factor, and BW, DIM, and grazing time (GT) as continuous covariates.

Rumination Time (Min/Day)						
Effect	Year-1		Year-2		Year-3	
	P	Var.	P	Var.	P	Var.
Breed	0.1605	2.19	0.1116	1.78	<0.0001	8.54
Lactation	0.2256	1.77	0.936	0.05	0.258	0.98
Cow (Breed*Lactation)	<0.0001	24.03	<0.0001	14.54	<0.0001	16.07
Season	<0.0001	2.31	<0.0001	16.98	<0.0001	7.41
Feed (Season)	<0.0001	1.70	<0.0001	2.11	<0.0001	1.64
Breed*Lactation	0.8699	0.71	0.725	0.79	0.6768	0.81
Breed*Season	<0.0001	0.12	0.0713	0.05	<0.0001	0.36
Lactation*Season	<0.0001	0.21	0.3961	0.02	0.0339	0.05
Breed*Lactation*Season	<0.0001	0.23	0.1147	0.08	<0.0001	0.62
Breeding worth (BW)	<0.0001	0.19	<0.0001	2.84	0.0305	0.02
Days in Milk (DIM)	<0.0001	3.31	<0.0001	1.75	<0.0001	2.98
Grazing time (GT)	<0.0001	5.54	<0.0001	2.17	<0.0001	2.14
BW*Breed	0.0084	0.05	0.0993	0.03	0.0309	0.03
DIM*Breed	0.0013	0.07	<0.0001	0.27	<0.0001	0.10
GT*Breed	0.0722	0.03	0.0959	0.03	0.6516	0.00
BW*Lactation	0.0011	0.07	<0.0001	0.12	<0.0001	0.09
DIM*Lactation	<0.0001	0.47	0.0014	0.08	0.0072	0.04
GT*Lactation	<0.0001	0.13	0.0385	0.04	0.1136	0.02
BW*Breed*Lactation	0.0056	0.07	<0.0001	0.32	<0.0001	0.12
DIM*Breed*Lactation	<0.0001	0.24	0.0027	0.10	0.0089	0.06
GT*Breed*Lactation	0.3848	0.02	0.0024	0.10	0.0311	0.05
GT*BW	0.4776	0.00	0.0008	0.07	0.2159	0.01
GT*DIM	<0.0001	0.30	0.3699	0.00	0.1728	0.01
GT*Season	<0.0001	0.30	0.9713	0.00	<0.0001	0.12
Total variance (%)	-	43.76	-	44.35	-	42.40

(P represents the *p*-value for the level of significance and Var. represents the variance in grazing time explained by the individual effects and their interactions included in the model. The significance level of the *p*-value was set at 0.05. * indicates an interaction between study factors).

## Data Availability

Not applicable.

## References

[1] Phillips C., Hecheimi K. (1989). The effect of forage supplementation, herbage height and season on the ingestive behaviour of dairy cows. Appl. Anim. Behav. Sci..

[2] Dillon P. (2007). Achieving high dry-matter intake from pasture with grazing dairy cows. Frontis.

[3] Kilgour R.J. (2012). In pursuit of “normal”: A review of the behaviour of cattle at pasture. Appl. Anim. Behav. Sci..

[4] Brumby P. (1959). The grazing behaviour of dairy cattle in relation to milk production, live weight, and pasture intake. N. Z. J. Agric. Res..

[5] Gonçalves E.N., Carvalho P.C.d.F., Kunrath T.R., Carassai I.J., Bremm C., Fischer V. (2009). Plant-animal relationships in pastoral heterogeneous environment: Process of herbage intake. Rev. Bras. Zootec..

[6] Newman J., Parsons A., Penning P. (1994). A note on the behavioural strategies used by grazing animals to alter their intake rates. Grass Forage Sci..

[7] Veerkamp R., Dillon P., Kelly E., Cromie A., Groen A. (2002). Dairy cattle breeding objectives combining yield, survival and calving interval for pasture-based systems in Ireland under different milk quota scenarios. Livest. Prod. Sci..

[8] Waghorn G., Clark D. (2004). Feeding value of pastures for ruminants. N. Z. Vet. J..

[9] Illius A. (1989). Allometry of food intake and grazing behaviour with body size in cattle. J. Agric. Sci..

[10] Funston R.N., Kress D., Havstad K., Doornbos D. (1991). Grazing behavior of rangeland beef cattle differing in biological type. J. Anim. Sci..

[11] Carvalho P.d.F. (2013). Harry Stobbs Memorial Lecture: Can grazing behavior support innovations in grassland management. Trop. Grassl..

[12] Arnold G., Fraser A.F. (1985). Association and social behaviour. Ethology of Farm Animals.

[13] Doyle P.T., Devendra C., Pearce G.R. (1986). Rice Straw as a Feed for Ruminants.

[14] Heublein C., Dohme-Meier F., Südekum K.-H., Bruckmaier R., Thanner S., Schori F. (2017). Impact of cow strain and concentrate supplementation on grazing behaviour, milk yield and metabolic state of dairy cows in an organic pasture-based feeding system. Animal.

[15] Harb M., Campling R. (1985). Variation among pregnant, non-lactating dairy cows in eating and ruminating behaviour, digestibility and voluntary intake of hay. Grass Forage Sci..

[16] Beauchemin K. (2018). Invited review: Current perspectives on eating and rumination activity in dairy cows. J. Dairy Sci..

[17] Sowell B., Mosley J., Bowman J. (1999). Social behavior of grazing beef cattle: Implications for management. Proceedings of the American Society of Animal Science.

[18] Nørgaard P., Nadeau E., Randby Å. (2011). A new Nordic structure evaluation system for diets fed to dairy cows: A meta analysis. Modelling Nutrient Digestion and Utilisation in Farm Animals.

[19] Schirmann K., Chapinal N., Weary D.M., Heuwieser W., Von Keyserlingk M.A. (2012). Rumination and its relationship to feeding and lying behavior in Holstein dairy cows. J. Dairy Sci..

[20] Ginane C., Baumont R., Favreau-Peigné A. (2011). Perception and hedonic value of basic tastes in domestic ruminants. Physiol. Behav..

[21] Llonch P., Mainau E., Ipharraguerre I.R., Bargo F., Tedó G., Blanch M., Manteca X. (2018). Chicken or the egg: The reciprocal association between feeding behavior and animal welfare and their impact on productivity in dairy cows. Front. Vet. Sci..

[22] Albright J. (1993). Feeding behavior of dairy cattle. J. Dairy Sci..

[23] Ginane C., Bonnet M., Baumont R., Revell D.K. (2015). Feeding behaviour in ruminants: A consequence of interactions between a reward system and the regulation of metabolic homeostasis. Anim. Prod. Sci..

[24] Bao J., Giller P., Kett J. (1992). The effect of milk production level on grazing behaviour of Friesian cows under variable pasture conditions. Ir. J. Agric. Food Res..

[25] Huzzey J., DeVries T., Valois P., Von Keyserlingk M. (2006). Stocking density and feed barrier design affect the feeding and social behavior of dairy cattle. J. Dairy Sci..

[26] Vijayakumar M., Park J.H., Ki K.S., Lim D.H., Kim S.B., Park S.M., Jeong H.Y., Park B.Y., Kim T.I. (2017). The effect of lactation number, stage, length, and milking frequency on milk yield in Korean Holstein dairy cows using automatic milking system. Asian-Australas. J. Anim. Sci..

[27] Lambert M., Litherland A. (2000). A practitioner’s guide to pasture quality. Proc. N. Z. Grassl. Assoc..

[28] Romanzin A., Corazzin M., Piasentier E., Bovolenta S. (2018). Concentrate supplement modifies the feeding behavior of Simmental cows grazing in two high mountain pastures. Animals.

[29] Sheahan A., Kolver E., Roche J. (2011). Genetic strain and diet effects on grazing behavior, pasture intake, and milk production. J. Dairy Sci..

[30] O’Driscoll K., Gleeson D., O’Brien B., Boyle L. (2010). Effect of milking frequency and nutritional level on hoof health, locomotion score and lying behaviour of dairy cows. Livest. Sci..

[31] Al-Marashdeh O., Gregorini P., Edwards G. (2018). Brief Communication: Effect of timing of maize silage supplementation on grazing behaviour of dairy cows during a short grazing session on a ryegrass-based herbage. N. Z. J. Anim. Sci. Prod..

[32] Kamphuis C., DelaRue B., Burke C., Jago J. (2012). Field evaluation of 2 collar-mounted activity meters for detecting cows in estrus on a large pasture-grazed dairy farm. J. Dairy Sci..

[33] Meagher R.K., Weary D.M., von Keyserlingk M.A. (2017). Some like it varied: Individual differences in preference for feed variety in dairy heifers. Appl. Anim. Behav. Sci..

[34] Berckmans D. (2014). Precision livestock farming technologies for welfare management in intensive livestock systems. Rev. Sci. Tech..

[35] Hostiou N., Allain C., Chauvat S., Turlot A., Pineau C., Fagon J. (2014). L’élevage de précision: Quelles conséquences pour le travail des éleveurs. INRA Prod. Anim..

[36] Andriamandroso A., Bindelle J., Mercatoris B., Lebeau F. (2016). A review on the use of sensors to monitor cattle jaw movements and behavior when grazing. Biotechnol. Agron. Société Environ..

[37] Henriksen J., Weisbjerg M., Løvendahl P., Kristensen T., Munksgaard L. (2019). Effects of an individual cow concentrate strategy on production and behavior. J. Dairy Sci..

[38] Munksgaard L., Weisbjerg M., Henriksen J., Løvendahl P. (2020). Changes to steps, lying, and eating behavior during lactation in Jersey and Holstein cows and the relationship to feed intake, yield, and weight. J. Dairy Sci..

[39] Prendiville R., Lewis E., Pierce K., Buckley F. (2010). Comparative grazing behavior of lactating Holstein-Friesian, Jersey, and Jersey× Holstein-Friesian dairy cows and its association with intake capacity and production efficiency. J. Dairy Sci..

[40] Grant R., Albright J. (2000). Feeding behaviour. Farm Animal Metabolism and Nutrition.

[41] Meisser M., Deléglise C., Freléchoux F., Chassot A., Jeangros B., Mosimann E. (2014). Foraging behaviour and occupation pattern of beef cows on a heterogeneous pasture in the Swiss Alps. Czech J. Anim. Sci..

[42] Demment M.W., Peyraud J.L., Laca E.A. Herbage Intake at Grazing: A Modelling Approach. Proceedings of the 4th International Symposium on the Nutrition of Herbivores.

[43] National Institute of Water and Atmospheric Research (NIWA) (2021). Annual Climate Summary. https://niwa.co.nz/sites/niwa.co.nz/files/2021_Annual_Climate_Summary_NIWA11Jan2022.pdf.

[44] Gregorini P., Minnee E., Griffiths W., Lee J. (2013). Dairy cows increase ingestive mastication and reduce ruminative chewing when grazing chicory and plantain. J. Dairy Sci..

[45] Dairy N.Z. Feed Requirements for Lactating Cows. https://www.dairynz.co.nz/feed/nutrition/lactating-cows/.

[46] Machado C., Morris S., Hodgson J., Fathalla M. (2005). Seasonal changes of herbage quality within a New Zealand beef cattle finishing pasture. N. Z. J. Agric. Res..

[47] Dairy N.Z. Supplementary Feed. https://www.dairynz.co.nz/feed/supplements/supplementary-feed/.

[48] Iqbal M.W., Draganova I., Morel P.C., Morris S.T. (2021). Validation of an Accelerometer Sensor-Based Collar for Monitoring Grazing and Rumination Behaviours in Grazing Dairy Cows. Animals.

[49] Müller R., von Keyserlingk M.A. (2006). Consistency of flight speed and its correlation to productivity and to personality in Bos taurus beef cattle. Appl. Anim. Behav. Sci..

[50] Villalba J.J., Provenza F.D., Catanese F., Distel R.A. (2015). Understanding and manipulating diet choice in grazing animals. Anim. Prod. Sci..

[51] Boval M., Sauvant D. (2019). Ingestive behaviour of grazing ruminants: Meta-analysis of the components of bite mass. Anim. Feed. Sci. Technol..

[52] Litherland A., Lambert M. (2007). Factors Affecting the Quality of Pastures and Supplements Produced on Farms. Occas. Publ.-N. Z. Soc. Anim. Prod..

[53] Poppi D., Hughes T., L’huillier P. (1987). Intake of pasture by grazing ruminants. Livest. Feed. Pasture. Hamilt. N. Z. Soc. Anim. Prod..

[54] Lambert M., Clark D., Grant D., Costall D. (1986). Influence of fertiliser and grazing management on North Island moist hill country 2. Pasture botanical composition. N. Z. J. Agric. Res..

[55] Hill J., Chapman D., Cosgrove G., Parsons A. (2009). Do ruminants alter their preference for pasture species in response to the synchronization of delivery and release of nutrients?. Rangel. Ecol. Manag..

[56] Jochims F., Soares É.M., De Oliveira L.B., Kuinchtner B.C., Casanova P.T., Marin L., De Quadros F.L.F. (2020). Timing and duration of observation periods of foraging behavior in natural grasslands. Front. Vet. Sci..

[57] DeVries T., Von Keyserlingk M., Weary D., Beauchemin K. (2003). Measuring the feeding behavior of lactating dairy cows in early to peak lactation. J. Dairy Sci..

[58] Bossen D., Weisbjerg M.R., Munksgaard L., Højsgaard S. (2009). Allocation of feed based on individual dairy cow live weight changes: I: Feed intake and live weight changes during lactation. Livest. Sci..

[59] Kertz A., Reutzel L., Thomson G. (1991). Dry matter intake from parturition to midlactation. J. Dairy Sci..

[60] Kidane A., Prestløkken E., Zaralis K., Steinshamn H. (2018). Effects of three short-term pasture allocation methods on milk production, methane emission and grazing behaviour by dairy cows. Acta Agric. Scand. Sect. A—Anim. Sci..

[61] Kröger I., Humer E., Neubauer V., Reisinger N., Zebeli Q. (2019). Feeding diets moderate in physically effective fibre alters eating and feed sorting patterns without improving ruminal pH, but impaired liver health in dairy cows. Animals.

[62] Litherland A., Woodward S., Stevens D., McDougal D., Boom C., Knight T., Lambert M. Seasonal Variations in Pasture Quality on New Zealand Sheep and Beef Farms. Proceedings-New Zealand Society of Animal Production.

[63] Lopes F., Coblentz W., Hoffman P., Combs D. (2013). Assessment of heifer grazing experience on short-term adaptation to pasture and performance as lactating cows. J. Dairy Sci..

[64] Wright M., Auldist M., Kennedy E., Dunshea F., Hannah M., Wales W. (2016). Variation in feeding behavior and milk production among dairy cows when supplemented with 2 amounts of mixed ration in combination with 2 amounts of pasture. J. Dairy Sci..

[65] Arachchige A.D.H., Fisher A.D., Auldist M.J., Wales W.J., Jongman E.C. (2013). Effects of different systems of feeding supplements on time budgets of cows grazing restricted pasture allowances. Appl. Anim. Behav. Sci..

[66] Moallem U., Altmark G., Lehrer H., Arieli A. (2010). Performance of high-yielding dairy cows supplemented with fat or concentrate under hot and humid climates. J. Dairy Sci..

[67] Sutherland T., Dobson A., Dobson M.J. (1988). Particle separation in the forestomachs of sheep. Aspects of Digestive Physiology in Ruminants.

[68] Aikman P., Reynolds C., Beever D. (2008). Diet digestibility, rate of passage, and eating and rumination behavior of Jersey and Holstein cows. J. Dairy Sci..

[69] Wilson W.C. (2017). Comparative Evaluation of the Behaviour of Holstein-Friesian and Jersey Cows under European Pasture-Based Management Systems. Masters Thesis.

[70] Rook A., Hopkins A. (2000). Principles of foraging and grazing behaviour. Grass: Its Production and Utilization.

[71] Grandl F., Luzi S.P., Furger M., Zeitz J.O., Leiber F., Ortmann S., Clauss M., Kreuzer M., Schwarm A. (2016). Biological implications of longevity in dairy cows: 1. Changes in feed intake, feeding behavior, and digestion with age. J. Dairy Sci..

[72] Dado R., Allen M. (1994). Variation in and relationships among feeding, chewing, and drinking variables for lactating dairy cows. J. Dairy Sci..

[73] Amaral-Phillips G.M.A.D.M. Merits of Having First Lactation Dairy Cows in a Separate Management Group. https://afs.ca.uky.edu/files/meritsofhavingfirstlacationdairycowsintwogroups.pdf.

[74] Konggaard S., Krohn C. (1978). Performance of First-Calf Heifers in Two Different Grouping Systems. Rep. Nat. Inst. Anim. Sci..

[75] Grant R., Albright J. (2001). Effect of animal grouping on feeding behavior and intake of dairy cattle. J. Dairy Sci..

[76] Maekawa M., Beauchemin K., Christensen D. (2002). Chewing activity, saliva production, and ruminal pH of primiparous and multiparous lactating dairy cows. J. Dairy Sci..

[77] Bowman G., Beauchemin K., Shelford J. (2003). Fibrolytic enzymes and parity effects on feeding behavior, salivation, and ruminal pH of lactating dairy cows. J. Dairy Sci..

[78] Fulkerson W., McKean K., Nandra K., Barchia I. (2005). Benefits of accurately allocating feed on a daily basis to dairy cows grazing pasture. Aust. J. Exp. Agric..

[79] Al-Marashdeh O., Gregorini P., Maxwell T.M., Cheng L., Beltrán I.E., Hussein A.N., Chen A., Guinot L., Hodge S., Cameron K.C. (2020). Short-term grazing and urination behaviour of dairy cows differing in their genetic merit. N. Z. J. Agric. Res..

[80] Rossi J. (2005). Differences in Grazing Behaviour and Herbage Intake between Genotypes and Holstein-Friesian Dairy Cows Grazing Short or Long Swards. Proceedings-New Zealand Society of Animal Production.

[81] Cazzuli F. (1999). Autores: Fiorella Cazzuli. Rev. INIA.

